# Accepting to Participate in an Early‐Phase Clinical Trial in Oncology: A Qualitative Study on the Patients' Experiences, Understanding, and Inner Motivations

**DOI:** 10.1002/pon.70291

**Published:** 2025-10-02

**Authors:** Estelle Guerdoux, Anne Stoebner, Louise Coutant, Marion Zamith‐Chavant, Sylvie Dolbeault, Jessica Martinez, Marta Jarlier, Diego Tosi

**Affiliations:** ^1^ Department of Palliative and Supportive Care Montpellier Cancer Institute University of Montpellier Montpellier France; ^2^ Desbrest Institute of Epidemiology and Public Health INSERM University of Montpellier Montpellier France; ^3^ Department of Supportive Care Toulouse Cancer University Institute ‐ Oncopole Toulouse France; ^4^ Department of Supportive Care Curie Institute Paris France; ^5^ SHARE PSL University Paris France; ^6^ Department of Medical Oncology Early Clinical Trial Unit Montpellier Cancer Institute University of Montpellier Montpellier France; ^7^ Biometrics Unit Montpellier Cancer Institute University of Montpellier Montpellier France; ^8^ Fondazione Gianni Bonadonna Milan Italy

**Keywords:** acceptation, cancer, clinical trial, coping, decision‐making, early‐phase, enrollment, experience, motivation, qualitative

## Abstract

**Background:**

The clinical trial landscape in oncology has evolved, with shifting endpoints and increased emphasis on early‐phase clinical trials (EPCTs) across European centers.

**Aims:**

This study aimed to update knowledge on the experiences of patients enrolling in EPCTs, focusing on the individual/contextual factors influencing decision‐making, and how to improve ethical and psycho‐oncological support.

**Methods:**

This qualitative single‐center study was part of a broader multicenter project. Twenty‐five patients with locally advanced or metastatic solid cancer, for whom standard therapies had failed, were interviewed face‐to‐face, at a French EPCT center. A reflexive thematic analysis of the transcripts was conducted, following Braun and Clarke's six‐step approach.

**Results:**

Four overarching themes emerged: “Experiencing the EPCT proposal”, “Accepting the EPCT”, “Ambivalent feelings” and “Coping strategies”, and several subthemes. Decisions were often made quickly in crisis situations and perceived as the only option. While patients reported receiving adequate information, their understanding of the trial's aims varied. Emotional distress and time pressure likely hindered full comprehension. Patients relied on trust in clinicians and reframed trial participation as a constructive goal, drawing on coping strategies to manage uncertainty and restore a sense of coherence.

**Conclusions:**

Patients were neither delusional nor misinformed, but their decisions were shaped by complex emotional and relational dynamics. The perception of being well informed does not ensure true understanding, especially in high‐stress contexts. Ambivalent emotions, tensions between expectations and hopes, and constrained choices should be considered when tailoring supportive care. Evidence‐based strategies are needed to enhance communication and shared decision‐making in EPCT settings.

## 1 Introduction

### 1.1 Background

Clinical trials (CTs) are essential for advancing medical care by assessing the effectiveness and safety of new interventions and treatments. Cancer treatments follow a developmental pathway that involves testing and implementation in which therapeutic efficacy is confirmed or not by CTs. Early‐phase CTs (EPCTs), also known as Phase 1 and 2 trials, represent the initial step in testing new drugs.

Understanding patients' experiences when deciding to participate in a CT after failure of standard therapies is essential to address ethical challenges and provide tailored supportive care. Recent study and systematic reviews have identified common factors influencing trial acceptance [1, 2, 3, 4]. Patients enrolling in Phase 1 trials are often motivated by hope for therapeutic benefit, shaped by cultural narratives of staying positive and trusting medical experts [1]. Their primary motivations include hope for cure, extended survival, and improved quality of life relative to the side effects [4], while altruism often appears as a secondary motivation. The decision‐making process also involves managing information needs, fears about trial participation, and a moral sense of contributing to research progress [2, 3, 4, 5, 6]. Trust in the physician's recommendation is consistently emphasized [1, 2, 3, 4, 7, 8]. Typically, the choice to consider trial participation follows cancer progression, with patients experiencing fear, anxiety, and psychological distress [2, 4]. Many describe participation as a “last resort,” intertwined with hope, existential fears, and the confrontation with mortality [2, 4].

The existing studies highlight the psycho‐oncological concerns of patients to whom a CT is proposed. Most studies have focused specifically on Phase 1, 2, or 3 CTs, while others have combined all phases, despite their distinct clinical and psychological implications. Specifically, Phase 3 CTs typically compare survival or side effects between treatment arms and are initiated only after phases 1 and 2 have established safety and tolerability. In contrast, EPCTs ‐ which include phases 1 and 2‐ raise specific psycho‐oncological challenges related to uncertainty, therapeutic hopes, and decision‐making under vulnerability. Furthermore, EPCT designs and endpoints are evolving [9]: traditionally intended to assess safety and dosing, some Phase 1 trials now incorporate preliminary efficacy endpoints, particularly in immuno‐oncology [10]. This evolution may alter patients' expectations and engagement with such trials.

Escritt et al.‘s systematic review [1] and Yang et al.‘s qualitative study [4] both provide valuable insights into hope, coping, and meaning‐making. However, they focus exclusively on Phase 1 trials and were conducted primarily in non‐European contexts (North America and Asia), where cultural norms, healthcare systems, and financial structures differ markedly from those in Europe. Also, the predominance of male participants in the qualitative study [4] may have influenced the findings given potential gender differences in coping and decision‐making. Additionally, some reviews have included participants who were offered but ultimately declined trial enrollment, without distinguishing their experiences from those of enrolled patients [2]. Lastly, in France, 16 centers were labeled “EPCT Centers” by the National Institute of Cancer in 2015 and 2019, with dedicated structures, monitoring systems, and financial support, fundamentally transforming access to innovation and patient care.

Taken together, these elements highlight the need for updated, context‐ and culture‐sensitive research on the experiences of patients facing an EPCT offer, particularly in real‐life European settings and within a healthcare system that guarantees financial coverage. The present study addresses this gap by qualitatively exploring the emotional, cognitive, and relational processes involved in the decision to participate in a Phase 1 or 2 trial in a French EPCT‐labeled center.

### 1.2 Aims and Objectives

The aim of this study was to update the qualitative understanding of the experience leading to acceptance of an EPCT by cancer patients. The primary objective was to identify the individual/contextual factors and enablers that influence decision‐making by patients with advanced cancer who accept to participate in an EPCT. Topics included the context, experiences, motivations and representations of the EPCT.

## 2 Methods

### 2.1 Ethics

This qualitative monocenter study was part of a broader multicenter project called “VRAIMENT” targeted on the patients' experience at the EPCT end. The study followed the Good Clinical Practice guideline and the Declaration of Helsinki, was registered at ClinicalTrials.gov (NCT03905876), and approved by the French national Human Ethics Committee (CPP Ref:2018T2‐15). The informed consent forms were revised and validated by a regional patient‐partner committee not linked to the investigator center. Verbal and written informed consents were obtained before the interviews. The authors followed the COREQ reporting guidelines [11].

### 2.2 Participants and Recruitment

Eligible participants were adults (≥ 18 years), with histologically confirmed locally advanced or metastatic solid cancer that did not respond to standard treatments, planned to receive antitumor treatment within an EPCT (Phase 1 or 2), with sufficiently fluent French to adequately complete the questionnaires, who signed the informed consent before any study‐specific procedures, and were affiliated to the French social security system or an equivalent scheme. Non‐inclusion criteria were: Montreal Cognitive Assessment test (MoCA) [12] score < 15/30, uncontrolled psychiatric disorders (excluding mood disorders in reaction to the disease) or treatment with discernment‐impairing psychotropics, available standard anticancer therapy, or enrolled in an EPCT the sponsor of which refused to integrate the VRAIMENT study. As suggested by the Braun and Clarke's reflexive thematic analysis approach [13], the purpose of patient recruitment and analysis was not to reach data saturation, but rather to deeply explore the patient experience and reasons for consenting to the EPCT. On the first day of the EPCT at the EPCT Center, patients were informed and offered the opportunity to also join the VRAIMENT study. If agreed, they were screened to ensure their eligibility.

### 2.3 Procedure and Data Collection

Interviews were conducted by EG (woman psycho‐oncologist with a PhD in psychology, neuropsychology graduate, and trained in mixed‐research methods) between January 2019 and June 2023. Inclusions were stopped during the COVID‐19 pandemia in 2020–2021. A semi‐structured interview guide was used (Table S1) that incorporated broad and open‐ended questions created by EG and AS (woman addiction medicine physician working in oncology, and trained in qualitative research), based on a review of clinically relevant issues identified in the EPCT literature and their previous clinical experience, and on discussions with DT (man oncologist, head of the EPCT Center) and some dedicated nurses (e.g., JM, woman nurse working at the EPCT Center). The questions covered EPCT context (perceived information and support), experience during the EPCT proposal, motivations to accept, and EPCT representation. Interviews took place face‐to‐face, in a quiet room inside the center, and lasted 31 min (± 7; range 11–38). Then, participants completed several self‐report questionnaires that are part of the VRAIMENT study protocol and were used here to describe the patients' clinical and psychological characteristics: Hospital Anxiety Depression Scale [14] (HADS) to determine the anxiety and depression levels, State‐Trait Anger Expression Inventory‐II [15] (STAXI‐II) to assess anger, EORTC QLQ‐C30 [16] to evaluate QoL, 10‐Item Connor‐Davidson Resilience Scale [17] (CD‐RISC‐10) to measure resilience, and Life Orientation Test‐Revised [18] (LOT‐R) to assess optimism.

### 2.4 Data Analysis

Interviews were audio‐recorded using a voice recording device and transcribed verbatim with a software (NVivo‐v11) and by an independent psychologist (not author) who corrected the transcribed data if necessary. The transcript reflexive thematic analysis was guided by Braun and Clarke's 6‐step approach. In step 1, EG read the transcripts and checked them against the original interview recordings to ensure accuracy and to become familiarized with the content. To prevent potential analysis biases, the primary coders (AS and LC) were blinded to the panrticipants' clinical status and motivations. In step 2, the initial coding of a subset of transcripts was carried out by EG, AS and LC. The analysts initially coded independently to ensure diverse viewpoints, then convened to discuss emerging codes and themes at 10 triangulation meetings (each 3 weeks) to determine whether they were in accordance with the identified codes. Divergent interpretations were neither automatically merged nor discarded but carefully considered and sometimes retained as subthemes or points of nuance, enriching the final thematic structure. Then (step 3), codes were categorized into four categories. In step 4 and 5, these four categories were refined into themes and formally defined at two supplementary meetings. The three analysts acknowledged that resolving discrepancies inherently involved their own perspectives, highlighting that theme development was an active and situated process of meaning‐making, rather than a neutral extraction of objective “truth”. Finally (step 6), they were summarized with extracts from the transcripts.

The sample characteristics were described by number of observations (*n*) and frequency (%) of each modality. The self‐report questionnaire scores were calculated in accordance with the relevant French guidelines [14, 15, 16, 17, 18], and described using median and extreme values (minimum and maximum) with the STATA v16.0 software.

## 3 Results

### 3.1 Sample Characteristics

For this qualitative study, 28 patients were approached and 25 accepted (response rate = 90%). Reasons for refusal were: fear of being judged (*n* = 2) and asthenia (*n* = 1). The characteristics of the 25 patients are listed in Table 1. All 25 participants (52% of women) were interviewed and completed the self‐report questionnaires (completion rate = 100%). Their median age was 61 years (range: 26–76), and the median time from diagnosis to enrollment was 2 years (range: 0.11–11.8). Six patients (24%) integrated a Phase 1 CT and 19 (76%) a Phase 2 CT. They were mostly married or de facto (60%, *n* = 15). Cancers concerned mainly the digestive tract (36%, *n* = 9), reproductive system (32%, *n* = 8), and lung (16%, *n* = 4), and metastatic disease was diagnosed in 80% (*n* = 20) of patients. The Eastern Cooperative Oncology Group Performance Status score was 1 in 83.3% of patients (*n* = 20). Patients had, on average, four symptoms (range 0–11), from grade 1 (in 54.2%, *n* = 13) to grade 3 (in 8.3%, *n* = 2). According to the MoCA scores (Table S2), global cognitive functioning was normal in 64% of patients (*n* = 16) [12]. Patients reported medium levels of resilience (CD‐RISC‐10 [17]) and optimism (LOT‐R [18]). Moreover, 72% (*n* = 18) of participants did not report any significant anxiety‐depressive symptom (HADS [14]) and 68% (*n* = 17) did not report anger (STAXI‐II [15]). The median QLQ‐C30 [16] score was 58 (range: 0–92 for Global health status); scores were higher for the “Fatigue,” “Pain,” “Dyspnea” and ‘Insomnia’ scales (median scores: 44, 33, 33 and 33, respectively).

**TABLE 1:** Sociodemographic and clinical characteristics of the study population.

Characteristics (*N* = 25)		
Sex^a^		
Females	13	(52)
Males	12	(48)
Age^a^	8	(32)
< 58 years [58–67] years	10	(40)
> 67 years	7	(28)
Marital status^a^		
Single, divorced, separated or widowed	10	(40)
Married or de facto	15	(60)
Professional status^a^		
Active	4	(16)
Retired	11	(44)
Sick leave	10	(40)
Education^a^		
Secondary school or below	11	(44)
Baccalaureate (high school diploma)	3	(12)
University degree	11	(44)
Place of residence^a^		
City	16	(64)
Rural	9	(36)
Primary tumor^a^		
Digestive (including colorectal)	9	(36)
Ovarian and reproductive system	8	(32)
Lung	4	(16)
Head and neck	1	(4)
Other	2	(8)
Unknown	1	(4)
Local recurrence^a^		
No	15	(60)
Yes	10	(40)
Metastasis^a^		
No	5	(20)
Yes	20	(80)
ECOG performance status^a^		
0	4	
1	20	(16.7)
Missing	1	(83.3)
Previous cancer treatments^a^		
Surgery	13	(52)
Chemotherapy	22	(88)
Radiotherapy	9	(36)
Targeted therapy	13	(52)
Hormonotherapy	2	(8)
Early‐phase clinical trial^a^		
Phase 1	6	(24)
Phase 2	19	(76)
Neuro‐psychological status^b^		
MoCA adjusted score	27	16–30]
HADS total score	11	6–27]
CD‐RISC‐10 total score	26	12–39]
LOT‐R total score	16	7–24]
STAXI‐II state score	15	14–49]

Abbreviations: CD‐RISC‐10 = 10‐Item Connor‐Davidson Resilience Scale; ECOG = Eastern Cooperative Oncology Group; HADS = Hospital Anxiety Depression Scale; LOT‐R = Life Orientation Test‐Revised; MoCA = Montréal Cognitive Assessment; STAXI‐II = State‐Trait Anger Expression Inventory‐II.

^a^ Values are expressed as number and percentage (%).

^b^ Expressed as median [range].

### 3.2 Overview of Themes

**FIGURE 1:**
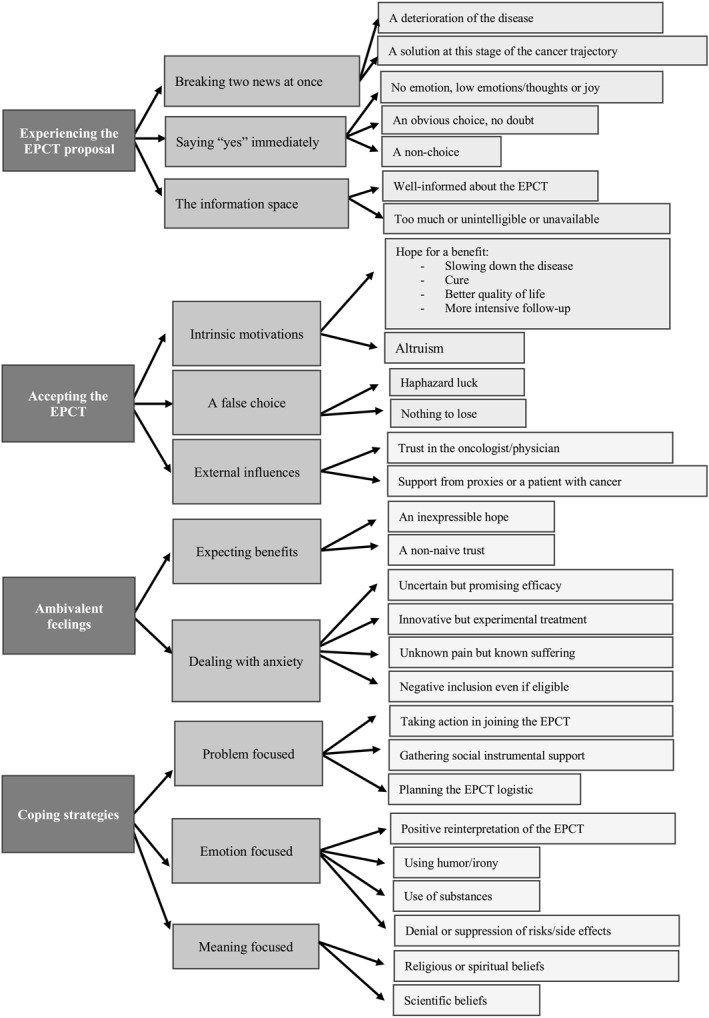
Summary of the thematic analysis results.

The analysis of the qualitative data resulted in four overarching themes: “Experiencing the EPCT proposal”, “Accepting the EPCT”, “Ambivalent feelings” and “Coping strategies”. Each theme contained subthemes that are summarized in Figure 1 and illustrated with quotes in Table 2.

**TABLE 2:** Patients' experiences, understanding, beliefs and motivations to join an early‐phase clinical trial in oncology.

Themes	Subthemes	Selected quotes
Experiencing the EPCT proposal	Breaking two news at once	*He [the oncologist] understood that his treatment had its limits and that something else must be done. He had the intelligence to suggest this trial to me.* (P. 119) *Shit, shit, shit, this is my home stretch […] But I'm so stupid, I'm benefiting from this, I've got a chance! Maybe not everyone does. If I was screwed, he [the oncologist] wouldn't make me do it.* (P. 02) *It's like mathematics… I was disappointed [with the results of the last scan] and since he [the oncologist] had… since he offered me this solution, well, it… it helped me regain… confidence, confidence, yes.* (P. 08)
	Saying “yes” immediately	*I immediately said yes.* (P.156) *I had no doubt, we must go ahead.* (P. 44) *For me, it seemed obvious to say yes.* (P. 22)
	The information space	*It was organized, it was structured, it was supported, it was explained* (P. 04) *I looked quickly in the evening because he [the oncologist] gave me a paper* (P. 03) *I know that she [the oncologist] said a lot of things, that we were full of it, and when we came out, we didn't know what it all meant…Each of us remembered little pieces of information, so we processed them to understand. They did give us a paper quite explanatory!* (P. 103)
Accepting the EPCT	Intrinsic motivations	*I said to myself: maybe there's hope*. (P. 11) *Honestly, this man inspired confidence. I will see him again next Wednesday. So, after that, I think he's good in his job. My life is in his hands, I mean.* (P. 119) *They're not trying to cure me! They're trying … to make sure it doesn't relapse again so quickly. To contain…It is to contain…She [the oncologist] told me I'll have a scan every 8 weeks…Well, following me more closely!* (P. 01) *If I will not be cured, well then, if I can retire in good conditions…I don't want to end up at the bottom of a bed! (…) bedridden…* (P. 03) *I benefit…from the research that has been done before me. Therefore, I am more than willing to help as much as I can…to help others who may be suffering, afraid…* (P. 13)
	A false choice	*I thought it was my last hope, that's what it was… I didn't have much choice. A slightly forced and coerced offer because… no other choice*. (P. 103) *I'm not going to say no, anyway, there's no other choice*. (P. 36) *Either we let ourselves die or… or else we do what … what they propose*. (P. 41)
	Extrinsic influences	*As I said to my oncologist: I'm putting myself in your hands, I don't know anything about it, I trust you completely.* (P.11) *Trust… Trust…It's just that they [oncologists] know that … that it wasn't…all for fun!* (P. 01) *So I'd say Professor X gave me confidence from the start*. (P.136) *My trust in Mr*. *Y is what gives me hope. Because I know he's doing his best… and I hope, in any case, in quotation marks, that… I even know that I trust him so much that I know he's not going to throw me… into something to hurt me! It's to… help me move on or get better. He's not going to do that to me! At least that's what I hope.* (P. 94)
Ambivalent feelings	Expecting benefits	*I'm pretty confident, knowing that I'm well aware that… oncology isn't the most exact science in the medical field! They're also testing their way through a lot of things and… But…I'm both confident and worried.* (P. 122) *Well, I'm not saying that I'm a guinea pig, but what I'm looking for in the story is for someone to treat me and tell me: ‘That's it, you're better now!’* (P. 18) *It's not because I think there's nothing left, there's no hope… It's quite the opposite! I think there are possibilities. At least, I think it'll be OK one day. I don't know…* (P. 01)
	Dealing with anxiety	*How should I know?! If I had two or three fellow patients who had the same thing as me, and who followed this protocol, who said to me, ‘Well, that will be OK’, then I'd be even more confident!”* (P. 41) *This kind of disease, it's not like the flu […] You never know! There's no date, there's no stop. It's the imaging that defines each treatment, the result. Living with uncertainty.* (P. 10) *For the moment, there's no time limit. What we do know… which is a certain stress factor for me, is… We don't know what the… the effects of the side effects, chemotherapy in particular…They are very different from person to person and we don't know in which cases… why one patient takes it well and another not at all. So, that worries me a bit*. (P. 22) *Well, I might feel nauseous, I might feel pins and needles, it's still the same system. First me as a guinea pig, then the others…* (P. 11) *Well, I get the heebie‐jeebies about what's going to happen. […]. Doctor X said ‘You're going to suffer’, well OK. I was warned that I was going to cop it. Like any trial, it can work and it can fail, hence the name*. (P. 04) *I don't know, we'll see, because they do many tests before…You have to accept it too!.. That's scary too, it's been giving me angsts since yesterday*. (P. 44)
Coping strategies	Problem focused	*I didn't inquire [about the EPCT] with the idea…to understand, to know everything […], but to have a little bit of control over something over which we have absolutely no control… I ask the doctors a lot of questions, I really try to understand. So, I keep constant surveillance.* (P. 22) *So, what do we do? Do we wait quietly for things to happen to us? No!* (P. 119)
	Emotion focused	*I'm very lucky to be treated here. That's not the case everywhere in France. So, not everyone is in the same boat. Here, we're lucky, we've got very, very good medicine, so there's no reason why we shouldn't put our trust in them.* (P. 22) *I feel like I've got myself and my cancer on the other side*. (P. 03) *For 3‐4 days, I was disturbed; I asked myself lots of questions and so on; and then it's back to normal. I went back to my routine, my everyday life, and … I forgot. I'm fine with that, but I think I'm perfectly normal.* (P. 119)
	Meaning focused	*The good Lord chose me. Well, listen, that's the way it is and that's the way it is.* (P. 11) *A lot of my hopes are pinned on uh …of my hopes, are pinned on the progress of…. research.* (P. 45) *I think that disease is not sent by God. There are a lot of factors that are part of an ordeal, that I have to work through…to overcome! And that I will overcome. My faith in God makes me think that I haven't come from far away to live here and that it's just going to end like this! I can't believe it!* (P. 13) *The innovative dimension… I've been following what's been going on in terms of cancer research today, and I can see how, since the Chicago congress, these questions of immuno, first of all, are at the heart of treatments. I'm very lucky to be treated in this city…there's very, very efficient science, so there's no reason not to trust them*. (P. 22).

### 3.3 Experiencing the EPCT Proposal

#### 3.3.1 Breaking Two News at Once

The decision to consider trial participation was reported as a reaction to cancer worsening. This situation was described as a rapid change in the health status or prognosis, a failure of the current treatment, or a change in a long medical history. Patients experienced feelings of deception or distress. During this consultation, the available therapeutic options, including the invitation to take part in the EPCT, were also introduced.

#### 3.3.2 Saying “Yes” Immediately

Due to the overwhelming emotions at this stage of their disease trajectory, most patients expressed no particular thought. They mostly felt no or low emotions, and accepted immediately the EPCT offer by their oncologist or their physician during a standard consultation. Three patients reported joy. For them, joy was not linked to hope for recovery but to the relief of having an option, of escaping passivity or emptiness, and of regaining a sense of action and direction in their care trajectory ‐ “*the end of the void*” (P.44), “*we can do something,*“ (P.136) or “*I still have options*” (P.156). In both case (no emotion or joy), patients reported no doubt, as if this was the obvious choice or a non‐choice.

#### 3.3.3 The Information Space

The interaction, information and exchange between patient and physician were globally adequate. Only few patients reported to be confused due to the excessive information received on the EPCT or by the specific jargon. Others read the written information after the consultation.

### 3.4 Accepting the EPCT

#### 3.4.1 Intrinsic Motivations

The primary motivation to enroll in the EPCT was the hope for a benefit (disease slow‐down or containment). Some participants hoped for a cure, whereas others expected a better QoL, a balance between longer life and side effects. The EPCT was also seen as an opportunity to have a more intensive follow‐up. Altruism was a secondary motivating factor, by envisaging that the trial may also help future patients and scientific advances, by contributing to better understand the disease.

#### 3.4.2 A False Choice

Patients reported to accept the enrollment in the EPCT as the only available option, leaving them with the sense that they had no real choice in the matter.

#### 3.4.3 External Influences

The clinicians' involvement was crucial in encouraging trial participation. They played a key role in their patients' decision‐making due to the deep trust established through the physician‐patient relationship. Patients considered that their oncologist had a profound understanding of their individual circumstances and profiles and that they were unlikely to recommend anything that could be non‐serious. The EPCT professionalism and rigor also influenced some decisions. Lastly, few patients mentioned the secondary influence of their family.

### 3.5 Ambivalent Feelings

#### 3.5.1 Expected Benefits

The analysis specifically differentiated between the participants' hopes and expectations about the EPCT. Most patients accepted that the EPCT would not treat/cure them, while at the same time they hoped this would happen. Participants were not delusional or misinformed; however, hope sometimes co‐existed with full awareness of the realities of their late‐stage disease. This incongruity was reflected by their ambivalent feelings and semantic oxymorons, as an inexpressible hope or a non‐naive trust.

#### 3.5.2 Dealing With Anxiety

Patients faced anxiety and fears. Common reasons for anxiety included the uncertain (but promising) efficacy of the new treatment, the side effects (feeling like a guinea pig) of the (innovative) treatment that could affect their quality of life, the potential pain (while having already experienced suffering), and the possibility of a non‐inclusion (despite an initial eligible profile for the EPCT).

### 3.6 Coping Strategies

Patients described a variety of strategies for coping with their EPCT acceptation during the waiting period before the trial start.

#### 3.6.1 Problem‐Focused Strategies

Most of the problem‐focused coping strategies included taking actions to manage the source of distress (i.e., cancer worsening) and to increase the feelings of control by accepting the EPCT. This included collecting information, gathering instrumental support and planning the EPCT logistic. Other patients reflected on their prior healthcare experiences to guide their future experience of a new cancer treatment.

#### 3.6.2 Emotion‐Focused Strategies

Patients described different emotion‐focused coping strategies, including positive reinterpretation of the EPCT, gathering emotional instrumental support, using a sense of irony, denying the risks of side effects, and suppressing negative emotions. Two patients used substances to face emotions.

#### 3.6.3 Meaning‐Focused Strategies

Some patients described using religion or spiritual beliefs to cope with their cancer worsening and participation in an EPCT. Scientific beliefs, such as faith in science, were also reported.

## 4 Discussion

This qualitative study update knowledges on patients experiences enrolling in EPCTs, complementing works by Escritt et al. [1] and Yang et al. [4]. First, we focused on the specific moment of trial proposal (*Remember when you were offered the clinical trial; what happened?*), a phase marked by urgency and emotional vulnerability, which remains underexplored in prior research, but also on the subsequent phase when the trial actually begins (*Where are you today with this clinical trial?*). Second, unlike previous studies limited to Phase 1 trials, our sample included patients enrolled in Phase 1 and 2 trials ‐ raising specific psycho‐oncological challenges and better reflecting the current landscape of CT ‐, and was gender‐balanced. Third, our study was conducted in a European setting with fully covered care, differing from mostly American or Asian contexts in previous studies [8].

The decision to join an EPCT was shaped by a complex interplay of emotional, psychological and practical factors arising from patients’ deteriorating health status and treatment failure. Trial offers coincided with acute crisis moments, leading to rapid, low‐deliberation decisions mostly driven by emotion rather than reflection. Patients reported minimal cognitive engagement, consistent with prior studies [2]. A minority expressed joy ‐not from unrealistic hope [4] but relief in re‐engaging with care. This re‐engagement was described as a source of relief and symbolic reassurance, as it restored a sense of being “cared for,” of being seen again by medicine. In this perspective, the trial was not merely a last therapeutic attempt, but a way to regain structure, legitimacy, and even dignity, at a time when standard care options had been exhausted. This re‐entry into an active care process, even under uncertain conditions, allowed some patients to mobilize adaptive coping mechanisms, fostering a renewed sense of coherence and purpose in the face of disease progression.

While prior research highlighted muddle about trial information [2, 5, 6, 7, 19], cognitive issues were less central here; 64% had a normal cognitive functioning, and most judged information adequate despite variable understanding of trial aims. Many initially felt trial participation was the only or obvious choice, reflecting an automatic emotional response. In a second step, patients cognitively reframed their decisions positively, aligning with Lazarus and Folkman's coping theory [20] and post‐decisional rationalization [21]. Primary appraisal relates to immediate emotional threat, and secondary appraisal involves re‐evaluation and meaning‐making in order to restore psychological balance. Similarly, the notion of “post‐decisional rationalization” [21] suggests that patients construct justifications and values after a decision, especially under high‐stakes or uncertain conditions, to reduce dissonance and consolidate their sense of agency.

Hope, distinct from expectation, was the main motivator, ranging from survival to QoL improvement. Participation was often perceived as the only option against worsening prognosis, mirroring Phase 1 or 3 trial [2, 3, 4] findings of “no real choice”. As previously shown [2, 4], trust in oncologists was crucial, convinced that they would not recommend non‐serious or harmful options. Patients valued personalized knowledge over detailed trial data.

Altruism was acknowledged but secondary, echoing Escritt et al. [1]. Unlike a systematic review [2] highlighting moral obligation, our participants did not feel normative pressure. Altruism, in this context, functioned more as a legitimizing discourse than a driving force. Family influence was minimal, possibly reflecting a coping strategy to maintain control and freedom amid uncertainty and loss of agency.

Psychological assessment showed medium optimism (LOT‐R^18^) and significant distress (HADS [14]) in 28% of patients, with a mixture of hope and anxiety. As recently shown [3, 4], most participants understood that the EPCT was not intended to cure them; however, they still hoped that it might. Consistent with previous studies on Phase 1 CTs [1, 4, 7, 22, 23, 24, 25], hope can exist alongside a clear understanding of the terminal illness realities. This discrepancy between expectations and hope can be a key motivator for participating in trials.

As previously reported [2], symptoms impacted QoL, with fatigue, pain, dyspnea, and insomnia prevalent (QLQ‐C30 [16]). Common concerns included additional potential side effects (both known and unknown), uncertain eligibility and efficiency, pain, feeling like a “guinea pig,” isolation. Participants used a variety of coping strategies to deal with anxiety and uncertainties. These ranged from problem‐focused approaches (been active, gathering information and planning logistics) to emotion‐focused and meaning‐focused coping strategies (emotional support, spiritual beliefs, or faith in science). This is consistent with the Sense of Coherence framework [26], which is considered to be part of the concept of resilience, and reflects the individual's resistance facing the stress with comprehensibility, manageability and meaning [27]. It emphasizes the need to understand the whole person rather than only focusing on specific issues or diagnoses. Patients should be seen as integrated beings (biological, psychological, social, and spiritual) who are both proactive and reactive, making choices and taking an active role in their disease. Additionally, salutogenesis views tension and stress as potential contributors to mental health rather than as inevitably harmful.

### 4.1 Clinical and Research Implications

Improving patient understanding and consent remains essential. Certification of EPCT centers may have positively impacted patient support by providing rigorous frameworks, monitoring, and logistical and financial security. Involving patient committees in reviewing consent documents is essential to ensure language reduces anxiety and feelings of vulnerability (e.g., being a “*guinea pig*”, facing “*strange*” acronyms). Trust in physicians is central to decision‐making, requiring clinicians to deeply understand each patient's context (i.e., past and current situation) beyond providing tailored trial information.

It is still crucial to distinguish hope from expectation when discussing EPCTs [1, 4]. While hope remains vital for coping with terminal illness, clinicians must balance realism with fostering hope, recognizing that meaning and hope can also arise from palliative care and existential support. Addressing supportive and palliative care options may reduce patients' sense of having “no choice” or a “nothing to lose” mentality and counteract biases toward active treatment. Grounded in the Sense of Coherence framework, we advocate promoting patients' coping resources and adaptation rather than solely focusing on risk reduction. Healthcare professionals should be trained to understand diverse coping strategies, several motivations, and temporalities of adaptation, including tolerance for ambivalence.

We also recommend developing adaptive, multimodal patient education tools co‐designed with patients to enhance comprehension and emotional engagement. Training programs for clinicians and research staff should include communication skills for managing ambivalence and differentiating hope from expectation, incorporating simulations and role‐plays [28]. Early and formal integration of supportive care teams (i.e., psycho‐oncologists and palliative care specialists) can address psycho‐emotional and existential needs throughout the trial. Patient and caregiver involvement via co‐construction groups and peer‐support can foster empowerment. Personalized longitudinal follow‐up using electronic patient‐reported (e‐PRO) outcomes can enable timely identification of distress or misunderstanding. Assigning a dedicated clinical research associate as a consistent contact ‐who would be more than just a “coordinator” [4]‐ can foster trust and ensure the clinic‐research link. Lastly, adopting comprehensive communication frameworks, such as Libert et al.‘s Advance Care Planning model with the CERTAIN mnemonic [29], can support clinicians in navigating uncertainty and sustaining hope while discussing complex EPCT issues. Continuous evaluation and quality improvement should accompany implementation to optimize ethical and psychosocial support in EPCT settings.

### 4.2 Study Limitations

This study was based on a careful design, including rigorous inclusion criteria and a validated quality assessment to determine the validity of the qualitative findings. However, it has some limitations. First, the small sample size may limit the ability to generalize findings to larger contexts and to replicate exactly the study. These limitations are inherent to qualitative research, and 25 participants may be considered a good sample size. Nevertheless, our results provide new themes, as well as they cover broad topics that were previously reported in different independent studies. Second, patient inclusion was interrupted during the COVID‐19 pandemic: potentially changing perception of temporality, vital urgency and QoL. Third, the interviewer's presence and behavior may have influenced the participants' responses and/or have introduced an awareness in the decision‐making process and coping strategies. Fourth, while many participants felt “well informed,” this perception must be interpreted with caution. Emotional distress can hinder understanding and memory, even in cognitively intact patients [30]. In our study, adequacy of information may have reflected a sense of being supported rather than a clear grasp of the trial's aims. Given that several participants described inclusion as the “only” choice, emotional and cognitive constraints likely limited reflective decision‐making. Without objective measures of comprehension, it is difficult to support the conclusion that communication and decision‐making support have improved. Lastly, due to the overlapping stresses at this cancer stage, it was difficult to distinguish the coping strategies used to face disease progression, EPCT decision‐making and uncertainties.

## 5 Conclusion

This qualitative research offers an updated understanding of how patients in France, an European oncology context, experience the decision to participate in EPCTs. Decisions were primarily driven by complex emotional, cognitive and situational factors, often made rapidly in crisis situations. While many patients reported receiving adequate information, content complexity was often eclipsed by the importance of trust‐based, individualized relationships with clinicians. Patients were neither misinformed nor delusional, but held on to hope for benefit while navigating uncertainty.

Coping strategies reflected efforts to reduce anxiety and restore a sense of coherence. Many patients expressed ambivalent emotions, balancing hope and realism ‐a tension clinicians and patients alike must learn to tolerate. Supporting this process requires communication strategies that are both ethically sound and evidence‐based.

Future studies should further investigate the quality of communication and decision‐making support by assessing the amount and type of information provided, evaluating patients' actual understanding and retention (e.g., brief assessments, patient reformulation), and examining whether time pressure affects decision quality. The role of these early decision‐making conditions and patient perceptions should also be explored throughout the trial trajectory, including final stages.

## Author Contributions

E.G. and D.T. designed the study and implemented it. E.G., A.S., J.M. and D.T. developed the interview guide. E.G. collected the patients' data. M.J. performed the descriptive statistical analyses. E.G., A.S. and L.C. performed the qualitative analyses. E.G. drafted the first manuscript, A.S., S.D. and L.C. drafted and revised the manuscript critically for important intellectual content. All authors, including M.Z.‐C., critically reviewed and approved the latest version for submission.

## Conflicts of Interest

E.G. declares no disclosure, relevant or directly related to the work. She reported grants from the French National Cancer Institute (Grant INCa_15779), from the Fondation de France, “Grant 2018, number 89251”, and from the SIRIC Montpellier Cancer (Grant INCa‐DGOS‐INSERM‐ ITMO Cancer_ 18004), during the conduct of the study, outside the submitted work. AS has no conflict of interest to declare and reported grants from *Groupement Interregional de Recherche Clinique Et d’Innovation* (GIRCI‐ AAP APIRES 2024), PHRIP (PHRIP‐15‐0526, NCT 03979924, PHRIP‐20‐0265, NCT 04457895) during the conduct of this study, outside the submitted work. L.C., M.Z.‐C., S.D., J.M., M.J. and D.T. have no conflict of interest to declare.

## Supporting information


Supporting Information S1

## Data Availability

De‐identified participant data will be made available upon request. Only requests that have a methodologically sound proposal will be considered. Proposals should be directed to the corresponding author; to gain access, data requestors will need to sign a data access agreement.
