# Supplementary material for: Accepting to Participate in an Early‐Phase Clinical Trial in Oncology: A Qualitative Study on the Patients' Experiences, Understanding, and Inner Motivations
Source: Psychooncology. 2025 Oct 2;34(10):e70291. doi: 10.1002/pon.70291 (PMC12491123; doi:10.1002/pon.70291)
Supplement: Supplementary file 1 — Supporting Information S1 [file PON-34-e70291-s001.docx]

**Supplementary Table 1.** Semi-structured interview guide.

| **Topics** | **Questions** |
| --- | --- |
| **Support and information**  [The question explores the context in which the clinical trial was proposed to the patient.] | ***To start, in what context were you proposed the clinical trial you are participating in?***  *Who offered you the clinical trial and where?*  *What does it involve?*  *What is its purpose?* |
| **Experiences**  [The question explores the patient experiences when the trial was proposed.] | ***Remember the moment when you were offered the clinical trial; what happened?***  *What did you think?*  *What did you feel?*  *What did you do?* |
| **Motivations**  [The question explores the decision-making processes.] | ***What were the reasons that led you to participate in this clinical trial?***  *What do you think made you agree to the trial?*  *How did you receive this trial proposal?* |
| **Representations**  [The question invites patients to summarize their experience, perceptions, understanding.] | ***Where are you today with this clinical trial?***  *What do you think of this early-phase clinical trial proposal in hindsight?* |

**Supplementary Table 2.** Results of the self-report questionnaires.

| Characteristics (N=25) | n^†^ | % |
| --- | --- | --- |
| Neurocognitive global functioning (MoCA)  Adjusted score <26 (neurocognitive impairment)  Adjusted score ≥26 (normal neurocognitive functioning) | 9  16 | (36)  (64) |
| Emotional status (HADS total score)  ≤14 (no anxiety-depressive symptoms)  >14 (anxiety-depressive symptoms)  Anxiety sub-score  <7 (no anxiety)  [8-10] (suspected anxiety)  [>10] (anxiety)    Depression sub-score  <7 (no depression)  [8-10] (suspected depression)  [>10] (depression) | 18  7  17  3  5  20  2  3 | (72)  (28)  (68)  (12)  (20)  (80)  (8)  (12) |
| Anger status (STAXI-II)  State score  <15  [15-16]  >16  Trait score  <14  [14-19]  >19 | 1  16  8  8  10  7 | (4)  (64)  (32)  (32)  (40)  (28) |
| Resilience (CD-RISC-10 score)  <25  [25-30]  > 30 | 6  11  8 | (24)  (44)  (32) |
| Optimism (LOT-R score)  <15  [15-17)  > 17 | 8  10  7 | (32)  (40)  (28) |

*Notes*. ^†^ Values are expressed as numbers and percentages (%); MoCA = Montréal Cognitive Assessment; HADS = Hospital Anxiety Depression Scale; CD-RISC-10 = 10-Item Connor-Davidson Resilience Scale; LOT-R = Life Orientation Test-Revised; STAXI-II = State-Trait Anger Expression Inventory
